# A Novel Elastomeric UNet for Medical Image Segmentation

**DOI:** 10.3389/fnagi.2022.841297

**Published:** 2022-03-10

**Authors:** Sijing Cai, Yi Wu, Guannan Chen

**Affiliations:** ^1^Key Laboratory of Optoelectronic Science and Technology for Medicine of Ministry of Education, Fujian Provincial Key Laboratory of Photonics Technology, Fujian Normal University, Fuzhou, China; ^2^School of Electronic & Electrical Engineering and Physics, Fujian University of Technology, Fuzhou, China; ^3^Fujian Provincial Engineering Technology Research Center of Photoelectric Sensing Application, Fujian Normal University, Fuzhou, China

**Keywords:** Parkinson’s disease, medical image segmentation, convolutional neural network (CNN), elastomeric UNet, UNet

## Abstract

Medical image segmentation is of important support for clinical medical applications. As most of the current medical image segmentation models are limited in the U-shaped structure, to some extent the deep convolutional neural network (CNN) structure design is hard to be accomplished. The design in this study mimics the way the wave is elastomeric propagating, extending the structure from both the horizontal and spatial dimensions for realizing the Elastomeric UNet (EUNet) structure. The EUNet can be divided into two types: horizontal EUNet and spatial EUNet, based on the propagation direction. The advantages of this design are threefold. First, the training structure can be deepened effectively. Second, the independence brought by each branch (a U-shaped design) makes the flexible design redundancy available. Finally, a horizontal and vertical series-parallel structure helps on feature accumulation and recursion. Researchers can adjust the design according to the requirements to achieve better segmentation performance for the independent structural design. The proposed networks were evaluated on two datasets: a self-built dataset (multi-photon microscopy, MPM) and publicly benchmark retinal datasets (DRIVE). The results of experiments demonstrated that the performance of EUNet outperformed the UNet and its variants.

## Introduction

With the popularization of medical imaging technology, such as magnetic resonance imaging (MRI), computed tomography (CT), positron emission tomography (PET), ultrasonic imaging, digital pathology, and microscopy, medical imaging technology plays an important role in clinical medicine to emerge the structure of human organs, tissues, and cells (Taruttis and Ntziachristos, 2015; Papadopoulos et al., 2017; Pang et al., 2020). The morphology of *in vivo* cells is an intuitive reflection of cell structure and function, and the analysis of it is a significant part of histopathology and clinical medicine to carry out clinical medical diagnosis and genetic research on aging, cell, and tissue expression of Alzheimer’s and Parkinson’s diseases (Li et al., 2010; Haft-Javaherian et al., 2019; Ye et al., 2020). Research on aging morphology and molecular mechanism of skin cells (especially *in vivo* skin cells) is an effective means for clinical medical research, such as skin aging and refractory skin wounds, and can provide the basis for aging cell research of a variety of aging-related diseases (e.g., cardiovascular disease, osteoporosis, and Parkinson’s disease) (Loeser, 2009; Aspray and Hill, 2019; Sun, 2020). Cortical angiography was used to explore the influences of Alzheimer’s disease and cellular senescence on capillaries (Haft-Javaherian et al., 2019). The incidence of melanoma in patients with Parkinson’s disease was studied by multiphoton imaging tracking, the dynamic process of melanocytic lesions (Balu et al., 2017; Lentsch et al., 2019; Ye et al., 2020). Image segmentation is an active research topic within medical image technology, which accounts for 70% of the international image processing competitions. As a basilic step in the medical image process, medical image segmentation separates the medical image into different regions with analogous characteristics to segment the regions of interest (ROI) and extract interrelated features. The role of medical image segmentation is to improve the intuitiveness of medical images and reduce man-made mistakes. Accurate medical image segmentation is difficult to achieve, owing to the data scarcity and image complexity, such as noisy background and intensity in-homogeneity.

The broad classification of medical image segmentation technology is traditional segmentation, such as thresholds, morphological methods (Chen et al., 2015) and clustering techniques, and deep-learning segmentation rest on CNN. Although various traditional algorithms have been designed to segment the medical images, the accuracy of segmentation is still difficult to improve. The fully convolutional network (FCN) designed by Long et al., 2015 is an end-to-end pixel-wise segmentation with a 20% amelioration of mean IOU than 2012 (Long et al., 2015). On account of significant improvement in the field of medical image segmentation by deep CNN, diverse variants of CNN are employed to segment medical images. As the development of FCN, UNet was applied to biomedical image segmentation by symmetric U-shaped architecture and skip connections yielding more accurate properties (Ronneberge et al., 2015). The improvement of UNet performance is apt to stagnate by the increase of depth. As a consequence, the diverse variants of UNet had been explored in the past 5 years for medical image segmentation (Li H. et al., 2018; Zhang et al., 2018; Liu et al., 2020). Inspired by the ResNet framework, a residual neural network with U-shape named U-ResNet was explored by Ibtehaz and Rahman (2020) and Drozdzal et al. (2016). In the same way, H-DenseUnet had been proposed to segment liver tumor mixed DenseNet to UNet by Li X. et al. (2018) and Cai et al. (2020). Another recent relevant work was Mask-RCNN, a novel UNet structure introduced by Vuola et al. (2019) through combining UNet and Mask-RCNN for nucleus segmentation. A U-shaped structure with dilated convolution path named Modified UNet was implemented by Zhao et al. (2019). UNet + + was employed to alleviate the *a priori* unknown of depth by Zhou et al. (2020), and the restrictive fusion architecture of UNet skip connections was also optimized in it. A UNet-like network entitled DSC-Net was performed to segment thymoma from CT images by Li et al. (2021). Another variant of UNet called X-Net was designed to segment liver and tumor by Chi et al. (2021). So far, however, the improved models still adopt a U-shaped structure, namely, the symmetrical single branch down-up sampling U-shaped structure. The performance of UNet is difficult to ameliorate, and the technical challenges of expanding the network size of UNet and extracting detailed information have prevented this architecture from being more accurate in medical imaging segmentation. Breaking the U-shaped architecture is an inevitable choice to overcome the technical bottleneck.

The purpose of this study was to design a flexible elastic expansion convolution operation to enhance the boundary detail extraction ability of the model and extend it to the segmentation of public medical image datasets by adjusting parameters through experiments. An elastomeric UNet (EUNet) has been proposed to address the limitations of U-shaped architecture, and the experimental validation demonstrates its effectiveness of it.

## Methodology

### UNet Model and Its Variants

**FIGURE 1:**
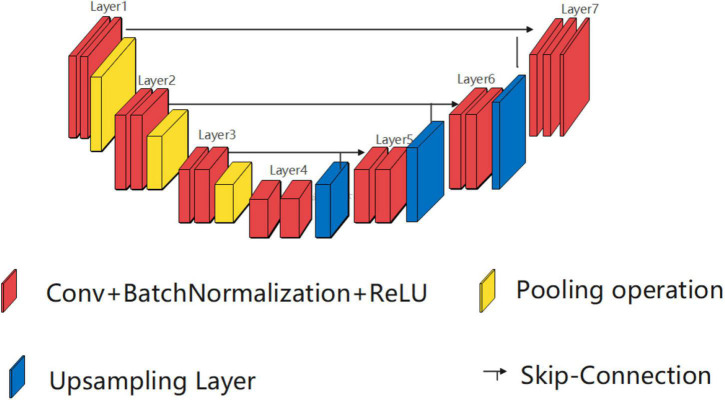
Structure diagram of UNet.

UNet was first proposed stemming from FCN. As shown in Figure 1, the U-shaped architecture of UNet is illustrated using three elements, namely, a contracting path, an expansive path, and skip connections. The key idea is to connect symmetric contracting path and expansive path by skip connections, where the convolutional operations are employed to extract features and upsampled operations are utilized to locate the ROI.

UNet + + is designed to overcome the two defects of UNet, *a priori* unknown of depth and the restrictive fusion architecture of skip connections. U-shapes with varying depths are integrated to relieve the *a priori* unknown of depth, and a pruning scheme is devised to accelerate convergence in UNet + +. Aggregation feature scheme instead of skip connections is executed in the up-sampling path to extract multi-scale features (Li X. et al., 2018).

Modified UNet extract varying scale features by introducing dilated convolution into U-shaped architecture (Cai et al., 2020). Each feature extraction operation consists of two steps. The first step is feature extraction operation through two convolution paths: one path is composed of ordinary convolution operation, and the other one uses dilated convolution for feature extraction. The second step is to complete feature fusion through a “concatenate” operation. A bigger receptive field is acquired in this way. Dilated convolution and conventional convolution coverage of receptive fields are the same when the dilate rate equals 1, the coverage goes to 5 × 5 when the dilate rate to 2 (Xia et al., 2020; Amer et al., 2021). Compared with the conventional convolution performed regular range feature extraction on fixed regions, the dilated convolution obtains larger receptive fields by the dilation rate.

### Proposed Model: Elastomeric UNet

The proposed model is built on the coding-decoding mechanisms, while novel structural breakthroughs are proposed over the original structure by adding a spatially extended network layer to improve the accuracy. Two types of EUNet, namely, horizontal EUNet and spatial EUNet, are introduced in this section, which include three extension structures of horizontal EUNet and two structures of spatial EUNet.

#### Horizontal Elastomeric UNet

Owing to the elastomeric wave horizontal transmission, the structure of horizontal EUNet was stretched horizontally by multiple feature extraction and spatial location algorithm to improve the segmentation accuracy. Horizontal EUNet expands the network by means of continuous U-shaped structure cascading and provides three structures according to the amounts of U-shaped structures that are cascaded: single-peak, double-peak, and three-peak horizontal EUNet. In the light of the position of wave peak, each structure is further characterized into two forms, i.e., low peak network and high peak network. There is less difference in performance between low peak and high peak networks, so this study introduces high peak networks.

##### Single High Peak Horizontal Elastomeric UNet

**FIGURE 2:**
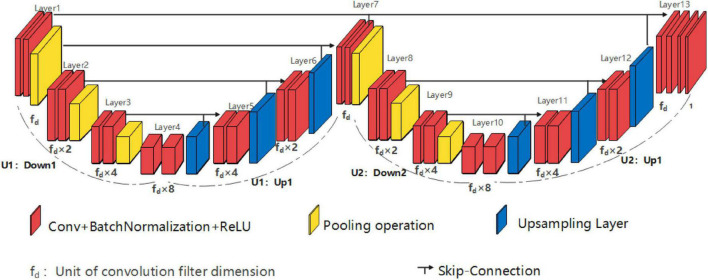
Structure diagram of single high peak horizontal EUNet (SHP-horizontal EUNet) U_1_(4-4)-U_2_ (4-4).

Single high peak horizontal EUNet (SHP-horizontal EUNet) contains a wave peak yielded W-shaped architecture. Due to twice consecutive down-up sampling, the feature extraction and space location operation were performed twice in the network to achieve more accurate pixel-to-pixel segmentation results. Figure 2 shows the structure of SHP-horizontal EUNet U_1_(4-4)-U_2_(4-4), which includes two symmetrical down-up paths forming a W-like structure. The input layer Lay_1_ and the output layer Lay_13_ of SHP-horizontal EUNet are located at both ends of the “W” shape on the same height and symmetrical to each other. The peak layer is also at the same height as the input/output layer. The outcome of the output layer is the cascaded output of the input/output layer and peak layer. There are two four-layered capture paths for feature extraction, indicating Down_1_ path (Lay1–Lay4) and Down_2_ path (Lay7–Lay10), and two extended paths for spatial information location, Up_1_ path (Lay4–Lay7) and Up_2_ path (Lay10–Lay13). Each down-layer is comprised of two convolutions to extract information and a pooling operation to reduce dimension for data compression, and each up-layer consists of a concatenate, an up-sampling, and two convolution operations. Batch normalization (BN) layers normalize the input value to improve the convergence of the gradient. Down_1_-Up_1_ form U_1_(4-4) structure, i.e., four layers of down-sampling and four layers of up-sampling, connected through the skip connections, and the structure of corresponding layers is completely symmetric. For instance, the resolution and dimension of Lay_2_ and Lay_6_ are the same, which is due to the skip connections. The network configuration is shown in Table 1.

**TABLE 1:** Architecture and parameters of single high peak horizontal elastomeric U-Net (SHP-horizontal EUNet).

Layer	Configuration of SHP-Horizontal EUNet	Feature size
Input	–	128×128

Layer1	f_*d*_×1, 3×3, Conv f_*d*_×1, 3×3, Conv 2×2, Pooling	128×128

Layer2	f_*d*_×2, 3×3, Conv f_*d*_×2, 3×3, Conv 2×2, Pooling	64×64

Layer3	f_*d*_×4, 3×3, Conv f_*d*_×4, 3×3, Conv 2×2, Pooling	32×32

Layer4	f_*d*_×8, 3×3, Conv f_*d*_×8, 3×3, Conv 0.5, Dropout	16×16

Layer5	f_*d*_×4, 2×2, Conv, 2×2, UpSampling Axis=3, concatenate f_*d*_×4, 3×3, Conv f_*d*_×4, 3×3, Conv	32×32

Layer6	f_*d*_×2, 2×2, Conv, 2×2, UpSampling Axis=3, concatenate f_*d*_×2, 3×3, Conv f_*d*_×2, 3×3, Conv	64×64

Layer7	f_*d*_×1, 2×2, Conv, 2×2, UpSampling Axis=3, concatenate f_*d*_×1, 3×3, Conv f_*d*_×1, 3×3, Conv 2×2, Pooling	128×128

Layer8	f_*d*_×2, 3×3, Conv f_*d*_×2, 3×3, Conv 2×2, Pooling	64×64

Layer9	f_*d*_×4, 3×3, Conv f_*d*_×4, 3×3, Conv 2×2, Pooling	32×32

Layer10	f_*d*_×8, 3×3, Conv f_*d*_×8, 3×3, Conv 0.5, Dropout	16×16

Layer11	f_*d*_×4, 2×2, Conv, 2×2, UpSampling Axis=3, concatenate f_*d*_×4, 3×3, Conv f_*d*_×4, 3×3, Conv	32×32

Layer12	f_*d*_×2, 2×2, Conv, 2×2, UpSampling Axis=3, concatenate f_*d*_×2, 3×3, Conv f_*d*_×2, 3×3, Conv	64×64

Layer13	f_*d*_×1, 2×2, Conv, 2×2, UpSampling Axis=3, concatenate f_*d*_×1, 3×3, Conv f_*d*_×1, 1×1, Conv	128×128

Output	2, 3×3, Conv 1, 1×1, Conv	128×128

*The second column illustrates the configuration of SHP-horizontal EUNet, and the third column denotes the output size of the feature map (MPM dataset).*

Researchers can flexibly adjust the structure of the network according to the characteristics of input pictures, such as using the SHP-horizontal EUNet U_1_(4-4)-U_2_(3-3). That is to say, the number of the sampling layer for different U-shaped structures is independent which means that researchers can determine how many layers are to be hired accordingly. For the dataset with image resolution between 128 and 600, it is recommended to adopt a small sampling structure with 4–5 layers. It is recommended to adopt a 4–5 layer sampling structure with a convolution filter equaled to (3 × 3). Generally, the below rules need to be followed:

➀ SHP-horizontal EUNet consists of two U-shaped structures. The input layer, wave peak layer, and output layer are at the same height.
➁ The down-sampling and up-sampling paths are completely symmetrical in each U-shaped structure, and different U-shaped structures are independent of each other, for example, there is no connection relationship between Lay2 and Lay8.
➂ The wave peak layer should have three groups of convolutional operations, namely, the cascaded input layer of the output layer, the output layer itself, and the output of the wave peak layer.

**FIGURE 3:**
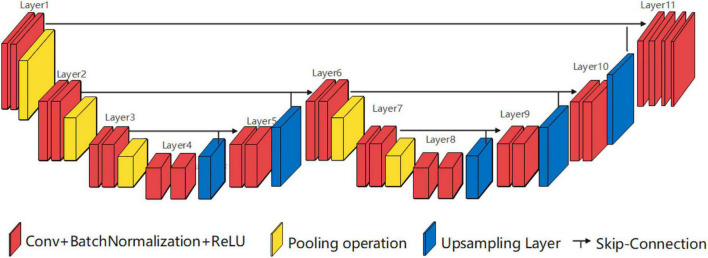
Structure diagram of single low peak horizontal EUNet (SLP-horizontal EUNet) U_1_(4-3)-U_2_(3-4).

Single low peak horizontal EUNet (SLP-horizontal EUNet) can also be designed, and the design principle is similar to SHP-horizontal EUNet. The input layer and the output layer of SLP-horizontal EUNet are also located at both ends of the “W” shape on the same height and symmetrical to each other. The difference between SHP- and SLP-horizontal EUNet is that the peak position is one layer lower than the input/output layer, as shown in Figure 3.

##### Double High Peak Horizontal Elastomeric UNet

**FIGURE 4:**
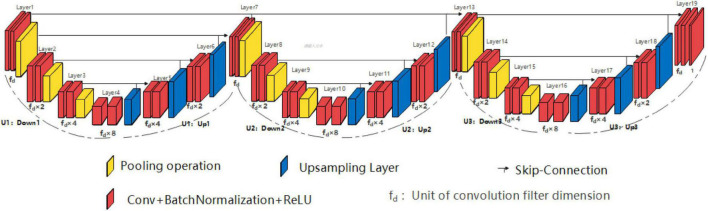
Structure diagram of double high peak horizontal EUNet (DHP-horizontal EUNet) U_1_(4-4)-U_2_ (4-4)-U_3_(4-4).

Similar to the SHP-horizontal EUNet structure, the double high peak horizontal EUNet (DHP-horizontal EUNet) is composed of three independent and fully symmetrical U-shaped structures by in-series connection. As shown in Figure 4, DHP-horizontal EUNet U_1_(4-4)-U_2_(4-4)-U_3_(4-4) is adopted in this section, which can be adjusted by the designer according to their needs, such as DHP-horizontal EUNet U_1_(5-5)-U_2_(3-3)- U_3_(4-4).

##### Three High Peak Horizontal Elastomeric UNet

**FIGURE 5:**
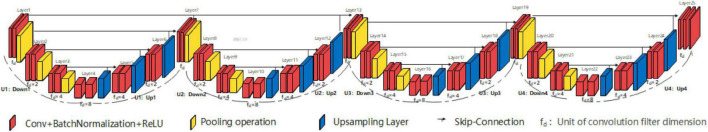
Structure diagram of three high peak horizontal EUNet (THP-horizontal EUNet) U_1_(4-4)-U_2_(4-4)-U_3_(4-4)-U_4_(4-4).

Consisting of four consecutive independent U-shaped structures, the three high peak horizontal EUNet (THP-horizontal EUNet) resembles SHP-horizontal EUNet structure which has four consecutive symmetrical U-shaped structures. As shown in Figure 5, different U-shaped structure is independent of each other. THP-horizontal EUNet U_1_(4-4)-U_2_(4-4)-U_3_(4-4)-U_4_(4-4) is exhibited in this section.

#### Spatial Elastomeric UNet

The spatial EUNet extends the network structure from both horizontal and vertical dimensions. The continuous cascade mode of horizontal EUNet is used on the horizontal dimension of spatial EUNet, and random seeds are used to generate multiple independent parallel branches on the vertical dimension. This design provides two spatial EUNet structures, which are double branches and three branches, denoting as U*UNet and U*U*UNet.

##### Double Branch Network: U*UNet

**FIGURE 6:**
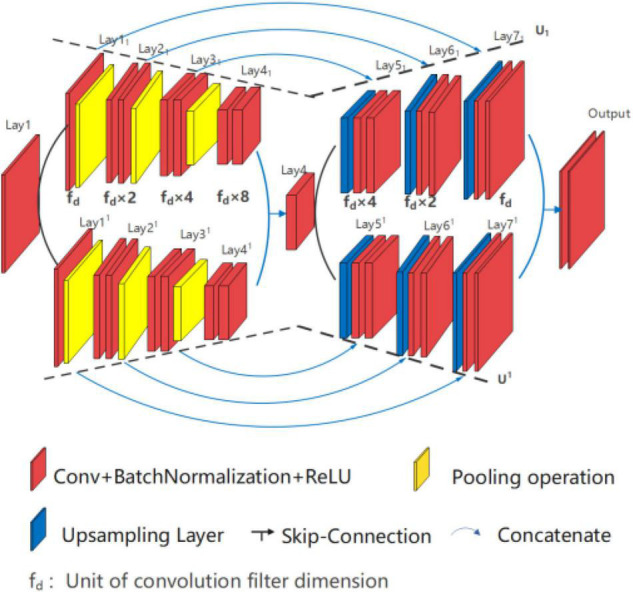
Structure diagram of U*UNet U_1_(4)//U^1^(4).

**FIGURE 7:**
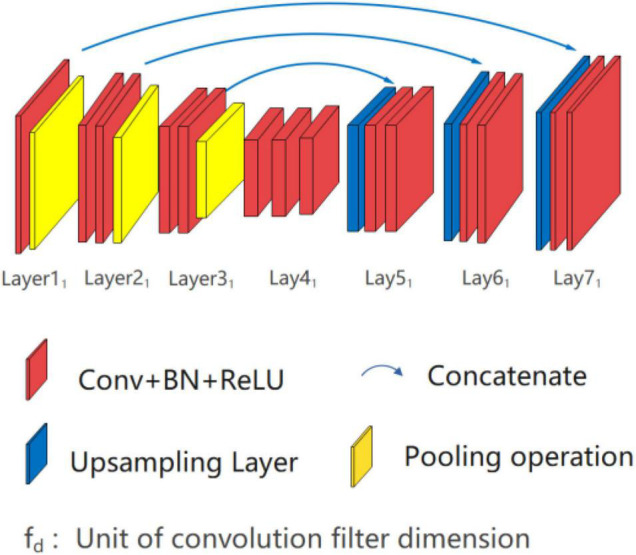
Schematic diagram of U_1_ path structure.

U*UNet, as shown in Figure 6, is composed of two U-shaped parallel U_1_ and U^1^, within Lay1_1_-Lay4-Lay1_7_ forms U_1_ path and Lay1^1^-Lay4-Lay1^7^ forms U^1^ path. Each path of U*UNet adopting full symmetric down-up sampling mode for feature extraction and location information recovery, such as the path U_1_, is shown in Figure 7. The separation operation is implemented in the input layer (Lay1) and peak layer (Lay4) to form two paths, and therefore, the convergence operation is executed at the valley (Lay4_1_ and Lay4^1^) and output layer (Lay7_1_ and Lay7^1^). Separation operation utilizes different random seeds for convolution operation, while merge operation is carried out for convergence. In addition, the two U-shaped paths are independent of each other, so multiform nesting, such as Densenet and Resnet, can be performed independently in each path on the basis of design requirements. This study does not do any nesting, but only provides the basic form of the running results.

The architecture design of U*UNet is as follows:

➀ U*UNet is composed of two in-parallel connected U-shaped structures.
➁ Each path contains an independent symmetrical U-shaped structure.
➂ The fuse output is realized through skip connecting *via* the wave valley and the output layer.

##### Three Branch Network: U*U*UNet

**FIGURE 8:**
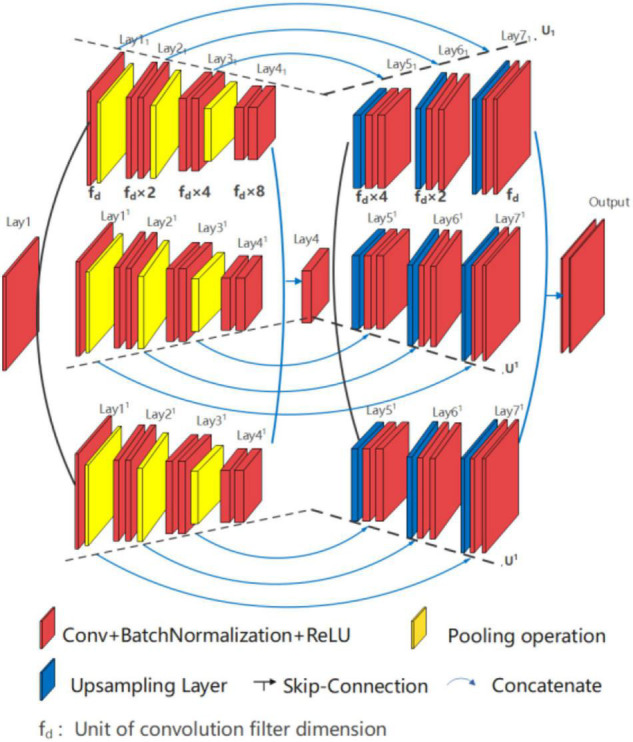
Structure diagram of U*U*UNet U_1_(4)//U^1^(4)//U^11^(4).

The structure of U*U*UNet is similar to U*UNet, with three U-shaped branches connected in parallel, as shown in Figure 8. Three branches are formed by convolutional division operation through the input layer (Lay1) and the wave valley (Lay4), on which the jump-connection aggregation is further taken place through the wave valley (Lay4_1_, Lay4^1^, and Lay4^11^) and the output layer (Lay7_1_, Lay7^1^, and Lay7^11^). Researchers can take other forms of nested superposition in different branches regarding different research targets.

**FIGURE 9:**
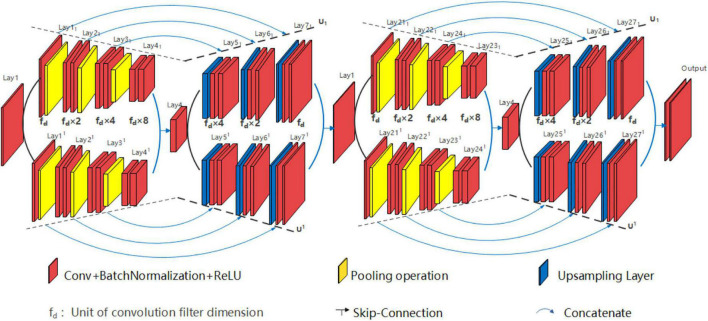
Structure diagram of signal peak U*UNet U_1_(4)//U^1^(4)-U_2_ (4)//U^2^ (4).

Various structures of EUNet can be employed, i.e., signal peak U*UNet U_1_(5)//U^1^(5)-U_2_(5)//U^2^(5) can be devised and signal peak U*UNet U_1_(3)//U^1^(3)-U_2_(5) can also be planned. For example, signal peak U*UNet U_1_(5)//U^1^(5)-U_2_ (5)//U^2^ (5) is shown in Figure 9. The signal peak U*UNet follows the design principle of horizontal EUNet in the horizontal direction with two consecutive down-up sampling structures. Its space expansion mode is the same as that of U*U Net. To sum up, the designer can design flexibly according to the dataset features, and design requirements for each path of EUNet are independent.

## Experiment and Results

### Dataset

All architectures proposed in the study were evaluated on two datasets, namely, the multi-photon microscopy (MPM) publicly benchmark retinal datasets (DRIVE). The self-built MPM imaging datasets were obtained by femtosecond home-testing Ti:Sa laser with a field view of 200 × 200 μm. An imaging system was carried out on Inc., Zeiss LSM 510 META with Plan-Neofluar objective. The testing data were taken from the dorsal forearm of the volunteer with a resolution of 128 × 128 pixels. Informed consent from each volunteer was acquired. The MPM dataset contains 90 images in.png format, of which 60 images are used for training, and the remaining 30 images are disposed of for testing. The DRIVE dataset contains 40 color retina images in.tif format, of which 20 images are used for training, and the remaining 20 images are disposed of for testing. The resolution of each image is 584 × 565 pixels at 45° field of view (Taruttis and Ntziachristos, 2015). The common medical image segmentation evaluation metrics suggested in the ISBI 2016 challenge (Ye et al., 2020) were used for benchmarking, including accuracy, precision, specificity, and mIOU. All networks were executed in Keras 2.24 with TensoFlow-GPU backend using Python 3.5.2. The training was implemented on a Microsoft Windows machine configured with GeForce GTX 1080Ti. All segmentation operations for two datasets were performed using the Dice coefficient loss function.

### Results

A series of experiments on MPM and DRIVE datasets was conducted to evaluate the performance between EUNet and U-shaped network (UNet, UNet + +, and Dilated UNet). The UNet and its variants were chosen because other deep network structures do not work well for the low resolution of MPM.

#### The Segmentation Results of the Multi-Photon Microscopy Dataset

In this section, the proposed EUNet was evaluated based on MPM. EUNet model adopts a 4-layered structure, for example, SHP-horizontal EUNet represents SHP-horizontal EUNet U1 (4-4)-U2 (4-4). The segmentation performance comparison between the proposed EUNet and the U-shaped network is summarized in Table 2, in which the segmentation results are also based on the accuracy, precision, specificity, and mIOU.

**TABLE 2:** Segmentation performance comparison between *EUNet* and U-shaped network in MPM dataset.

Method		Accuracy	Precision	Specificity	mIOU
UNet		88.90	92.69	84.85	77.90
UNet + + (L3)		88.59	92.01	83.67	77.41
UNet + + (L4)		88.10	91.30	82.08	76.49
Dilated UNet(L1)		88.33	94.20	88.79	77.42
Dilated UNet(L2)		87.52	90.06	79.11	75.29
Dilated UNet(L3)		87.35	89.92	78.81	74.99
Horizontal EUNet	SP- Horizontal EUNet	91.25	96.24	92.82	82.38
	DP-Horizontal EUNet	91.58	95.88	91.88	83.02
	TP- Horizontal EUNet	91.48	94.94	89.82	82.70
Spatial EUNet	U*UNet	91.54	94.66	89.19	82.77
	U*U*UNet	91.21	93.40	86.33	81.99

**FIGURE 10:**
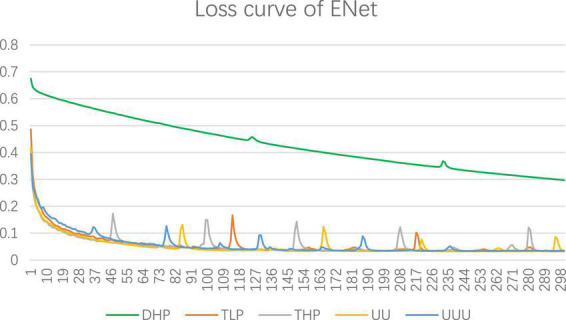
Segmentation loss curve for MPM dataset.

UNet + + (L3) and UNet + + (L4) represent three-layered and four-layered nested structures, respectively, while dilated UNet(L1) denotes that the top layer using dilated convolution instead of convolution and (L2) means that the top two layers employ dilated convolution in down-sampling, and so on. UNET + + and dilated UNet perform comparably of MPM dataset, which segmentation accuracy is lower than that of UNet. Dilation convolution is not effective in small resolution datasets, especially with the deepening of dilated convolution. The performance of EUNet proposed in this article all show improvements based upon the U-shape network, where the average accuracy, precision, Specificity, and mIOU achieve 91.41%, 95.02%, 90%, and 82.57% respectively, yielding average improvement of 2.51 and 4.67 points in accuracy and mIOU than UNet respectively. EUNet shows good convergence, as shown in Figure 10.

**FIGURE 11:**
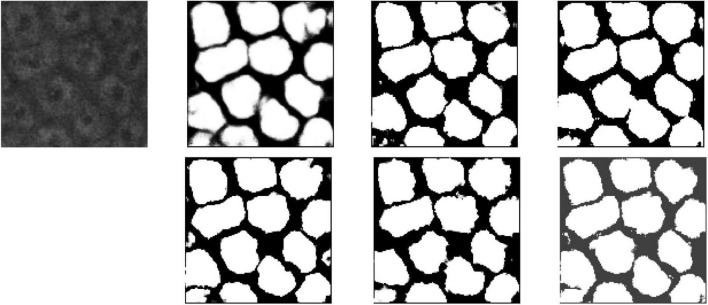
Segmentation results for MPM dataset. Row 1, column 2: UNet; row 1, column 3: UNet + + (L4); row 1, column 4: dilated UNet(L2); row 2, column 2: DHP-horizontal EUNet; row 2, column 3: U*UNet; row 1, column 4: U*U*UNet.

For a better understanding of performance for each model intuitively, segmentation results of UNet, DHP-horizontal EUNet, and U*UNet are illustrated in Figure 11. It can be seen from the figure that EUNet embodies better boundary segmentation by comparing the outline detail in the second, third, and fourth images. In addition, it is noteworthy that more accurate edge feature extraction is achieved by U*UNet.

#### The Segmentation Results of the DRIVE Dataset

In this section, EUNet and U-shaped networks were tested and verified based on the DRIVE dataset. Each EUNet model adopts a 5-layered structure, for example, SHP-horizontal EUNet represents SHP-horizontal EUNet U1 (5-5)–U2 (5-5). Table 3 shows the segmentation results that were assessed in the light of accuracy, precision, specificity, and mIOU.

**TABLE 3:** Segmentation performance comparison between EUNet and U-shaped network in DRIVE.

Method		Accuracy	Precision	Specificity	mIOU
UNet		95.97	81.20	98.28	79.15
UNet + + (L3)		93.41	61.42	95.12	72.30
UNet + + (L4)		94.57	72.42	97.43	73.60
Dilated UNet(L1)		94.11	73.99	98.02	70.14
Dilated UNet(L2)		94.16	76.51	98.35	69.69
Dilated UNet(L3)		93.88	75.24	98.33	68.26
Dilated UNet(L4)		92.89	65.71	97.48	65.36
Horizontal EUNet	SHP-Horizontal EUNet	96.15	83.62	98.56	78.75
	DHP-Horizontal EUNet	96.36	83.12	98.44	80.17
	THP- Horizontal EUNet	96.22	81.69	98.27	77.43
Spatial EUNet	U*UNet	96.48	85.42	98.71	79.79
	U*U*UNet	96.56	83.19	98.39	82.03

As seen, expansion networks bring as a result to significant improvement for DRIVE dateset, which with average improvement of 0.38 points over UNet. Among the space expansion networks, the performance of U*U*UNet is the best, the accuracy of U*U*UNet is up to 96.56%, which is higher than that of UNet 0.59%. The precision of U*UNet is 85.42%, which is higher than that of UNet 4.22%. Experimental results showed that the proposed EUNet provided good performance than UNet. Compared with the UNET in the DRIVE dataset, the Dilated UNet has reduced performance, and the average accuracy is reduced by 2.21 points, which is lower than that of EUNet by 2.59 points. The results show that expansion convolution performs better inmedium resolution datasets, such as DRIVE dataset.

**FIGURE 12:**
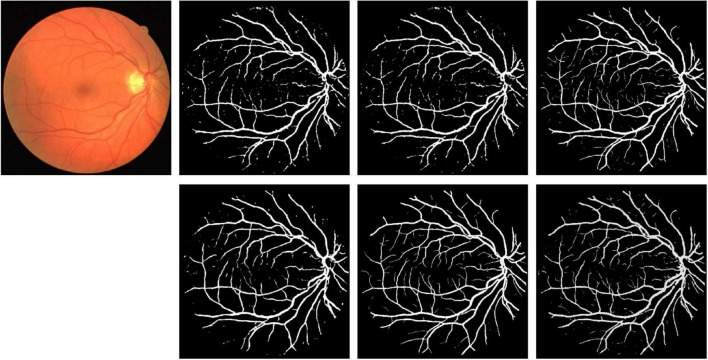
Segmentation results for DRIVE dataset. Row 1, column 2: UNet; row 1, column 3: UNet + + (L4); row 1, column 4: dilated UNet(L2); row 2, column 2: DHP-horizontal EUNet; row 2, column 3: U*Unet; row 1, column 4: U*U*UNet.

Partial segmentation results are illustrated in Figure 12. As shown in the figure, more details can be described by elastomeric networks. The best background noise processing ability is shown by U*UNet.

## Discussion

In general, the overall performance of MPM cell segmentation is limited by the amount of dataset and the resolution size. Based on preliminary work, this study provides EUNet. According to their different structural characteristics, each network embodies different segmentation characteristics, as follows:

In summary, the main contributions of this study include:

(1) We proposed EUNet for medical image segmentation. This network provides the possibility for the malleable development of networks through the horizontal and vertical extension of the elastomeric wave, which greatly breaks through the shackles of traditional structures.
(2) Each corresponding peak layer and valley layer of the branch are operated by fusion convolution in spatial EUNet to realize the multipath information complement and to overcome the position information loss caused by down-sampling.

(3) Every path in this study maintains relative independence, which provides flexible redundancy for researchers. Designers can flexibly design diverse structural variants based on image characteristics and the consideration of Res-Net and Dense-Net. For example, the researcher can carry out multi-form nesting in any U-shaped path regarding the design demand or choose different segmentation model parameters to adjust in different paths (e.g., Conv (3)-Conv (3) can be employed in one path, Conv (3)-Conv (1)-Conv (3) can be utilized in the other path in U*UNet, and so forth).

## Conclusion

A novel EUNet is presented in this study to overcome the fixed U-shaped structure of medical image segmentation, which is desirable for the research of Parkinson’s disease and gene aging. Multiple expansion-contraction paths are provided to extract edge detailed features. Convergence is executed to strengthen the fusion of local and global information in peak and valley layers of different branches. The datasets employed in this design were MPM and DRIVE datasets to verify the effectiveness of EUNet. The experimental results demonstrate that the EUNet all show good accuracy and robustness in two datasets. The detailed description is strengthened, and the multi-scale features are fully described by adding feature channels. The consecutive expand-contract path fully explores the dependency of context information and enhances the association feature representation of each semantic class to make the detailed expression of the image edge clearer. Moreover, the model provides a very flexible and extensible structure for future medical image segmentation owing to the relative independence of each U-shaped structure. Researchers can design each U-shaped structural level, functional structure, and parameter model independently according to the characteristics of the segmented images, which will further offer unprecedented room for segmentation resolution improvement.

## Data Availability Statement

The data analyzed in this study is subject to the following licenses/restrictions: this is the *in vivo* skin cell dataset. Requests to access these datasets should be directed to caisijing@163.com.

## Ethics Statement

The studies involving human participants were reviewed and approved by Fujian Normal University. The patients/participants provided their written informed consent to participate in this study. Written informed consent was obtained from the individual(s) for the publication of any potentially identifiable images or data included in this article.

## Author Contributions

YW: responsible for the overall planning of the project. SC: responsible for CNN model design, improvement and implementation, experimental test and data analysis. GC: responsible for providing experimental data and model design guidance. All authors contributed to the article and approved the submitted version.

## Conflict of Interest

The authors declare that the research was conducted in the absence of any commercial or financial relationships that could be construed as a potential conflict of interest.

## Publisher’s Note

All claims expressed in this article are solely those of the authors and do not necessarily represent those of their affiliated organizations, or those of the publisher, the editors and the reviewers. Any product that may be evaluated in this article, or claim that may be made by its manufacturer, is not guaranteed or endorsed by the publisher.
